# NHP BurkPx: A multiplex serodiagnostic bead assay to monitor *Burkholderia pseudomallei* exposures in non-human primates

**DOI:** 10.1371/journal.pntd.0011067

**Published:** 2023-02-08

**Authors:** Kimberly R. Celona, Austin B. Shannon, Derek Sonderegger, Jinhee Yi, Fernando P. Monroy, Christopher Allender, Heidie Hornstra, Mary B. Barnes, Elizabeth S. Didier, Rudolf P. Bohm, Kathrine Phillippi-Falkenstein, Daniel Sanford, Paul Keim, Erik W. Settles

**Affiliations:** 1 Pathogen and Microbiome Institute, Northern Arizona University, Flagstaff, Arizona, United States of America; 2 Department of Mathematics and Statistics, Northern Arizona University, Flagstaff, Arizona, United States of America; 3 Department of Biological Sciences, Northern Arizona University, Flagstaff, Arizona, United States of America; 4 Tulane National Primate Research Center, Tulane University, Covington, Louisiana, United States of America; 5 Battelle Memorial Institute, Columbus, Ohio, United States of America; USAMRIID: US Army Medical Research Institute of Infectious Diseases, UNITED STATES

## Abstract

**Background:**

Melioidosis is a disease caused by the bacterium *Burkholderia pseudomallei*, infecting humans and non-human primates (NHP) through contaminated soil or water. World-wide there are an estimated 165,000 human melioidosis cases each year, but recordings of NHP cases are sporadic. Clinical detection of melioidosis in humans is primarily by culturing *B*. *pseudomallei*, and there are no standardized detection protocols for NHP. NHP are an important animal model for melioidosis research including clinical trials and development of biodefense countermeasures.

**Methodology/Principle findings:**

We evaluated the diagnostic potential of the multiple antigen serological assay, BurkPx, in NHP using two sera sets: (i) 115 *B*. *pseudomallei-*challenged serum samples from 80 NHP collected each week post-exposure (n = 52) and at euthanasia (n = 47), and (ii) 126 *B*. *pseudomallei*-naïve/negative serum samples. We observed early IgM antibody responses to carbohydrate antigens followed by IgG antibody recognition to multiple *B*. *pseudomallei* protein antigens during the second week of infection. *B*. *pseudomallei* negative serum samples had low to intermediate antibody cross reactivity to the antigens in this assay. Infection time was predicted as the determining factor in the variation of antibody responses, with 77.67% of variation explained by the first component of the principal component analysis. A multiple antigen model generated a binary prediction metric ($\hat{p}$), which when applied to all data resulted in 100% specificity and 63.48% sensitivity. Removal of week 1 *B*. *pseudomallei* challenged serum samples increased the sensitivity of the model to 95%.

**Conclusion/Significance:**

We employed a previously standardized assay for humans, the BurkPx assay, and assessed its diagnostic potential for detection of *B*. *pseudomallei* exposure in NHP. The assay is expected to be useful for surveillance in NHP colonies, in investigations of suspected accidental releases or exposures, and for identifying vaccine correlates of protection.

## Author summary

Melioidosis is a disease caused by the soil-dwelling bacterium *Burkholderia pseudomallei*, a Tier 1 Select Agent by the US Centers for Disease Control and Prevention (CDC), with the ability to naturally infect humans and non-human primates (NHP). Although melioidosis diagnosis in humans has been formally outlined, there is no standardized test available for *B*. *pseudomallei* exposure and melioidosis in NHP. NHP are important animal models in melioidosis research in addition to other scientific research. The goal of this study was to formally evaluate the first extensive serodiagnostic test for detection of *B*. *pseudomallei* exposure in NHP. The BurkPx assay comprised 21 *B*. *pseudomallei* specific antigens and was previously standardized in humans. Early IgM antibody responses were noted to carbohydrates whilst IgG antibody responses were mounted to multiple proteins during the second week of infection. The assay elicited low background reactivity to *B*. *pseudomallei*-naive NHP. Data collected from the BurkPx assay was fed into a multiple antigen model, which produced a $\hat{p}$ score used to classify the assay via thresholding. The final model produced a 100% specificity and 63.48% sensitivity among all samples; however, removal of early infection samples increased sensitivity to 95%.

## Introduction

Melioidosis is an infectious disease well-known for its burden on humans in endemic regions since the early 1900s [1], but this illness is equally important for animals, including non-human primates (NHP). Melioidosis is caused by the intracellular pathogen *Burkholderia pseudomallei*, which is common in sub-tropical and tropical regions of the world; most noteworthy are Northern Australia and Southeast Asia [2]. Through percutaneous inoculation, inhalation, ingestion, or injection, humans and animals alike can become infected with *B*. *pseudomallei* after contacting contaminated soil or water [1,3]. Onset of symptoms can range from 1 to 21 days, with a median incubation of four days until presentation and an inter-quartile range (IQR) of 3–7 days [4]. Once infected, symptoms are protean, ranging from a flu-like illness to septicemic shock [3]. Humans who are immunocompromised and agricultural workers in endemic areas are at a higher risk for infection [3,5,6]. Recently, the estimated burden of human melioidosis cases has been predicted to be about 165,000 cases per year world-wide, with roughly 89,000 resulting in death [7].

The current gold standard for melioidosis detection in humans is to culture *B*. *pseudomallei* from a clinical specimen [1]. Culture methods result in high specificity (100%), but low sensitivity (~60%) [8]. Moreover, culturing often requires special laboratory infrastructure, such as a biosafety level 3 laboratory space to handle the pathogen. Turnaround time, sometimes taking up to seven days for positive culture, is a serious limitation for human diagnostics. *B*. *pseudomallei* is intrinsically resistant to many antibiotics and so treatment regimens are long and should be initiated early in the course of disease, if possible. Beyond culture methods, serological assays can complement culture diagnosis, with the standard test being the indirect hemagglutination assay (IHA). The IHA detects IgM antibodies in blood to *B*. *pseudomallei* whole cell lysates (WCL). While highly specific in non-endemic areas, the IHA is challenged by background or cross-reactive seropositivity in endemic regions, reducing the specificity while maintaining a ~56% sensitivity in any region [9,10]. Many other diagnostic tests have been evaluated such as lateral flow immunoassay, immunofluorescence assay, protein microarray, enzyme linked immunosorbent assay, and polymerase chain reaction, but no test has been widely distributed and approved for clinical use to replace culture [11].

Unlike for humans, there are few published data about disease burden in NHP, even though natural infections occur. However, a handful of reported melioidosis cases in NHP dating back to the 1920’s in Malaysia, Australia, United States of America (US), India, France, Britain, and Indonesia [12–15] suggest that the burden is sporadic. These cases consisted of disease in NHP in wild colonies, zoos, and primate research centers. Several of these outbreaks included NHP imported from endemic to non-endemic regions, which raises the potential of introducing *B*. *pseudomallei* into a new environment. Importation of *B*. *pseudomallei*-infected NHP is a serious issue in the US since *B*. *pseudomallei* is deemed a Tier 1 select agent by the US Centers for Disease Control and Prevention (CDC) [16]. A memo from the CDC sent to the Association of Primate Veterinarians in February 2022 stated that approximately 60% of NHP imported to the US originate in countries where melioidosis is endemic and *B*. *pseudomallei* is present in the environment. Detection of *B*. *pseudomallei* infection in NHP are frequently accomplished by culture and colony morphology and sometimes serology, but these tools are inadequate and often involve subjective interpretation since the protocols used are outlined for detection in humans. For example, the IHA has not been formally evaluated for *B*. *pseudomallei* detection in NHP, leaving a gap in the establishment of diagnostic criteria which determines disease versus non-disease status. NHP in outdoor environments come in contact with soil on a regular basis and may be exposed to various types of soil bacteria that can cause immune responses and thus false-positive IHA results [17]. These types of considerations need to be addressed when developing a diagnostic test for melioidosis in NHP.

The importance of NHP and melioidosis research lies in the interface of clinical trials and the development of countermeasures to potential biothreats. Studies on melioidosis vaccines are now guided by several regulations. The Food and Drug Administration (FDA) animal rule requires that vaccine benefits be reported in more than one animal species to enhance prediction of the likely response of humans [18]. In addition, the Steering Group Melioidosis Vaccine Development (SGMVD) states “efficacy studies in a non-human primate model may be required prior to advancement of the vaccine to human clinical trials” [19]. Although the cost of NHP trials is high, NHP are valuable animal models for vaccine studies due to their translational relevance to clinical melioidosis [20]. Several publications describe the use of NHP as an animal model for *Burkholderia spp*. [21–23]. In *Burkholderia* biodefense research, NHP have been used in studies to determine Lethal Dose-50 (LD_50_) metrics. The development of accurate diagnostic assays to screen NHP for previous or existing infection with *Burkholderia spp*. prior to enrollment as research subjects is critical to the success of these programs and increases rigor and reproducibility.

The purpose of this study was to formally evaluate the first extensive serodiagnostic test for detection of *B*. *pseudomallei* exposure in NHP. Rather than developing a new diagnostic test specifically for NHP, we implemented an established human BurkPx assay to test NHP serum samples. This assay has the potential to serve as a screening tool for imported and domestic colony born NHP to detect exposure to *B*. *pseudomallei*, as well as potentially monitor immune responses to animals enrolled in therapeutic vaccine studies for correlates of efficacy and protection.

## Methods

### Ethics statement

The animal samples used in this study were acquired in accordance with the Institute of Laboratory Animal Resources (ILAR) Guide for the Care and use of Laboratory Animals, the National Institutes of Health (NIH) Public Health Service Policy on the Humane Care and Use of Laboratory Animals. Samples were approved by the Battelle (approval number 2783) or the Tulane National Primate Research Center institutions Institutional Animal Care and Use Committee (IACUC).

### Animal use and serum collection

**Fig 1:**
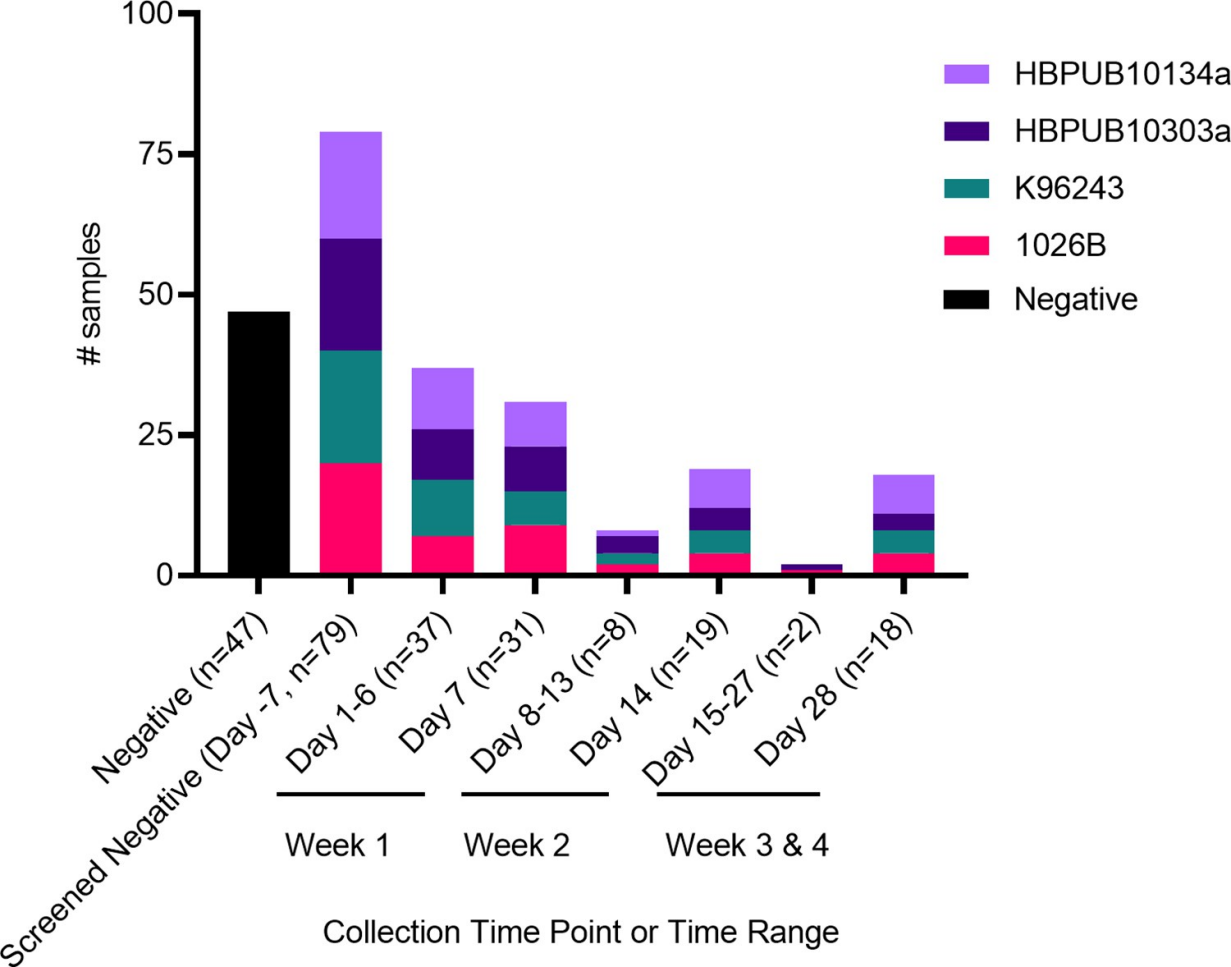
Distribution of *B*. *pseudomallei*-challenged and negative sera screened with the BurkPx assay. Forty-seven *B*. *pseudomallei* negative sera collected from routine cross-colony surveys of non-human primates at TNPRC were screened with this assay (black bar, first column) to serve as *B*. *pseudomallei* cross-reactivity controls. The distribution of serum samples (n = 115) collected from 80 rhesus macaques involved in the inhalational LD_50_ determination experiment are shown over time. Days pre- or post-challenge and total number of sera samples obtained at each time point are shown in parentheses. The different challenge strains of *B*. *pseudomallei* associated with each sample are indicated by the colored stacked bars.

Serum samples from NHP that were aerosol-challenged with *B*. *pseudomallei* (n = 115) were provided by Dr. Daniel Sanford at Battelle Memorial Institute (BMI) from an LD_50_ experiment [24]. Eighty (80) rhesus macaques imported from a Chinese origin and pre-screened with a *B*. *pseudomallei* WCL Enzyme Linked Immunosorbent Assay (ELISA) for reactivity were challenged via aerosol exposure to four different *B*. *pseudomallei* strains at the following target challenge doses (CFU/animal): 50, 100, 500, 100, 1,000, 10,000, 100,000, and 500,000 (S1 Table). Serum was collected from each animal 7 days before exposure, each week post exposure, and at the time of euthanasia (Fig 1). In this sample set, we only received 79 pre-challenge samples to screen. Animals evaluated for LD_50_ determination were observed for clinical signs, evaluated for body temperature three times a day, including weekends, and humanely euthanized once they met criteria for euthanasia.

Serum samples from NHP housed at Tulane National Primate Research Center (TNPRC) (n = 47) that were *B*. *pseudomallei* negative were provided by Dr. Richard Bohm. These samples were collected during semi-annual health assessments of NHP in the breeding colony in 2018. NHP were anesthetized with ketamine hydrochloride (10mg/kg IM) intramuscularly for blood collection. The purpose of the second set of negative samples was to obtain normally distributed *B*. *pseudomallei* cross-reactivity that may represent the true serological background of rhesus macaques in captivity.

All serum samples were aliquoted into small volumes and frozen at -80°C.

### Preparation of antigens and conjugation to microspheres

Antigen selection, purification, and conjugation have been described [25]. In short, 21 *B*. *pseudomallei* antigens, including recombinant proteins or purified carbohydrates, were conjugated to MagPlex microspheres. *B*. *pseudomallei* proteins were cloned in an *Escherichia coli* expression plasmid with a 6-histidine tag on the N-terminus of the protein. Insoluble proteins were solubilized chemically with the addition of N-Lauroylsarcosine [26]. Solubilized proteins were purified using a AKTA FPLC system with nickel column. All buffers contained N-Lauroylsarcosine to maintain solubility if used to solubilize the target. The inclusion of N-Lauroylsarcosine does not impact protein coupling to MagPlex microspheres since it does not have any amine groups [26]. Each purified protein was conjugated to different fluorescently labelled MagPlex microspheres using the two-step carbodiimide coupling reaction at pH 5.6 using: Sulfo-NHS (N-hydroxysulfosuccinimide) and EDC (1-ethyl-3-(3-dimethylaminopropyl) carbodiimide hydrochloride) (Luminex xMAP Cookbook, 2^nd^ Ed.). Naturally soluble proteins were conjugated with 5 μg protein per one million beads. In the presence of N-Lauroylsarcosine 48 μg protein was conjugated per one million beads. Successful conjugations were confirmed by detection of the 6-histidine tag by a monoclonal anti-6-histidine antibody (Abcam, ab15145) conjugated to biotin and examination on a MAGPIX assay. The purified carbohydrates, capsular polysaccharide (CPS) and lipopolysaccharide (LPS) were conjugated through activated carbohydrate (active ether) chemistry using 4-(4,6-dimethoxy-1,3,5-triazin-2-yl)-4-methyl-morpholinium chloride (DMTMM) [27]. For the conjugation, reactive carbohydrates with DMTMM were purified with PD-10 desalting column. Successful conjugations were confirmed using LPS or CPS specific monoclonal antibodies kindly provided by Dr. David AuCoin at the University of Nevada, Reno. Additionally, four controls, two for confirmatory antibody response (IgM and IgG), one to monitor machine response (PE), and the last as a negative control (pDEST TRX) were added to the bead set. A summary of the final bead set is shown in Table 1.

**Table 1:** *B*. *pseudomallei* proteins, carbohydrates, and assay controls comprising the BurkPx multiplex serological assay.

Antigen	Locus Tag	Sub-Localization^1^	Antigen Type
BPSS0135	BPSS0135	Unknown	Protein
GroEL	BPSL2697	Cytoplasmic	Protein
MSHR5855 WCL	Not Applicable	Not Applicable	Protein
BPSS1652	BPSS1652	Unknown	Protein
Arg	BPSL1743	Cytoplasmic	Protein
ClpX	BPSL1404	Cytoplasmic	Protein
rpIL	BPSL3222	Unknown	Protein
GroS	BPSS0476	Cytoplasmic	Protein
GroEL2	BPSS0477	Cytoplasmic	Protein
AhpC	BPSL2096	Cytoplasmic	Protein
DNAK	BPSL2827	Cytoplasmic	Protein
OmpA	BPSL2522	Outer Membrane	Protein
BPSS1850	BPSS1850	Outer Membrane	Protein
LPSA	Not Applicable	Outer Membrane^2^	Carbohydrate
LPSB	Not Applicable	Outer Membrane^2^	Carbohydrate
CPS	Not Applicable	Outer Membrane^3^	Carbohydrate
NADH	BPSS1769	Cytoplasmic Membrane	Protein
BPSS0530	BPSS0530	Cytoplasmic	Protein
HCP1	BPSS1498	Extracellular	Protein
IPMS	BPSL1201	Cytoplasmic	Protein
AtpD	BPSL3396	Cytoplasmic	Protein
IgM Positive Control	Not Applicable	Not Applicable	Not Applicable
IgG Positive Control	Not Applicable	Not Applicable	Not Applicable
PE Instrument Control	Not Applicable	Not Applicable	Not Applicable
pDEST TRX Stop Negative	Not Applicable	Not Applicable	Not Applicable

^1^PSORTb Subcellular Localization Predication Tool https://www.psort.org/psortb/.

^2^Perry, MacLean [28]

^3^Masoud, Ho [29]

### MAGPIX assay

One day prior to performing the MAPGIX assay, conjugated beads in Table 1 were mixed to achieve 1000 beads per region per 100 μL for one reaction. Beads were suspended in phosphate buffered saline (PBS) containing 1% Bovine Serum Albumin (BSA, Fisher P137525) to block the beads. Aliquots of 100 μL of bead mixture were dispensed into wells of a black polystyrene flat bottom, non-sterile 96-well plate (Grenier, M4926), and protected from light for the duration of the experiment. NHP serum samples were thawed, mixed, and diluted 1000-fold in 1% BSA in PBS. Each serum sample was screened in duplicate. MAGPIX assays were performed in a biosafety cabinet in a biosafety level 2 laboratory. Before application of serum, beads were washed two times with 200 μL of 1X PBS-T (0.05% Tween-20) using a plate washer (BioTek 405TS Microplate washer). Following the initial wash, 100 μL of diluted serum was added to wells containing beads and allowed to incubate for two hours at room temperature with shaking at 400 rpm. Following serum incubation, beads were washed three times with 200 μL 1X PBS-T. Washed beads were incubated with 2 μg/mL of antibody isotype and species-specific secondary antibody bound to biotin suspended in 100 μL of 1% BSA in PBS buffer per reaction (goat anti-monkey IgM (Sigma, SAB3700781); goat anti-monkey IgG (Fitzgerald, 43RIG021bt)). Reactions were incubated for one hour at room temperature with shaking at 400 rpm and then washed three times as before. Streptavidin, R-Phycoerythrin (SAPE, Life Technologies S866) was added to the beads at 4 μg/mL in 1% BSA in PBS. Reactive antibodies were quantified with a Luminex MAGPIX system resulting in median fluorescence intensity (MFI) values for each of the antibody bound to each target antigen. Data were evaluated for each microsphere region achieving at least 30 counts and if control values (i.e., IgG bead reacting in IgG assay) produced results in the standard range. An average MFI was calculated from the replicates and used in data analysis.

### Statistical analyses

Statistical analyses were performed using Graph Pad Prism 8 (Graph Pad Software Inc., La Jolla, CA). Receiver Operator Characteristic (ROC) curves were used to identify the sensitivity and specificity values from results of the multiple antigen models. Heat maps were generated from average MFI data for each sample and antigen. Box and whisker plots were presented with 95^th^ and 5^th^ percentile boundaries in the box with the median values shown as the line in the box. Additionally, Youden’s J Statistic was applied to ROC analyses to aid in cut-off determinations and to maintain a high specificity (100%) [30].

A Principal Component Analysis (PCA) was implemented to determine if antigen responses of sera between *B*. *pseudomallei*-challenged and *B*. *pseudomallei*-negative NHP displayed a temporal progression of variation due to duration of infection. Average median fluorescence intensity values (MFI) from the BurkPx assay for 115 *B*. *pseudomallei*-challenged and 126 *B*. *pseudomallei*-negative samples (79 pre-challenge samples from BMI and 47 samples from TNPRC, Fig 1) were imported into QIIME 2 software as a feature table [31]. Euclidean distance and Bray Curtis dissimilarity metric were evaluated and visualized with Emperor [31]. Both were chosen based on their suitability for handling multivariable, non-phylogenetic data. The Euclidean distance calculates the absolute difference between two points, observing the overall shape of the response. In contrast, the Bray Curtis dissimilarity quantifies the difference between two samples, taking into consideration abundance. Both measures were applied to produce a diversity matrix and subsequently used in conjunction with QIIME 2 principal coordinate plug-in to create a PCA. Visualization was performed using emperor plot and QIIME 2. A PCA was produced for IgM and IgG isotypes separately. A permutational analysis of variance (PERMANOVA) was used to determine significance between the independent TNPRC negative dataset and the time-series groups from the LD_50_ *B*. *pseudomallei* aerosol challenge study in the Euclidean distance matrix.

Two binomial regression models were developed to interpret the data between *B*. *pseudomallei*-challenged versus *B*. *pseudomallei*-negative NHP. The result of these multiple antigen models is a simplified binary called $\hat{p}$ probability score. The Ridge Regression method is a binomial logistic regression model with an additional term added to the likelihood that penalizes superfluous model complexity and pushing the regression coefficients towards (but not to) zero. Across all levels of the penalty tuning parameter, all antigens are included in this model, although their impact might be negligible. The LASSO method also starts with a binomial logistic regression with an additional penalty term, but modification forces the regression coefficients to zero as the penalty parameter term is increased, effectively sequentially removing covariates from the model. This modification allows the ranking of antigens and their specific immune response (IgG or IgM) that contribute to the model. In this model 10 antigens are in IgG LASSO, 7 antigens are in the IgM LASSO, and 22 antigens are in the IgGM LASSO (5 IgG only, 5 IgM only, 6 for both IgG and IgM, S2 Table). All model analyses were completed with glmnet package version 3.0–2 and R package version 3.6.0 [32,33].

To evaluate model capabilities, 58 *B*. *pseudomallei*-challenged, and 64 *B*. *pseudomallei*-negative samples were randomly chosen to build an independent test-set validation model. A naïve dataset of 57 *B*. *pseudomallei*-challenged, and 62 *B*. *pseudomallei*-negative samples were used to evaluate the model efficacy by receiver operator characteristic (ROC) plots and box and whisker plots. Using the same antigens as the independent test-set model, a second model was built with all the data of each group–i.e.– 115 *B*. *pseudomallei*-challenged samples and 126 *B*. *pseudomallei*-negative samples and performance of this second model was evaluated using cross-validation and threshold selection was optimized to require; (1) equal or better performance than the corresponding independent test-set model, (2) highest sensitivity in week 1, and (3) specificity must be set at 100% for comparisons.

To differentiate the species-specific (NHP) components of the BurkPx assay, including modification of the antibody and specific design of the model, the assay will be coined ‘NHP BurkPx’ in the remaining parts of the study.

## Results

### Antibody responses of *B*. *pseudomallei*-challenged and *B*. *pseudomallei*-negative rhesus macaques to BurkPx antigen set

Twenty-one antigens were selected for the BurkPx antigen assay, and these antigens were chosen through multiple antigen model analyses on their ability to differentiate between healthy human and melioidosis reactive individuals [25]. Here we evaluated the ability of the human targeted BurkPx antigen assay to detect exposure in NHP challenged with different *B*. *pseudomallei* strains. To this end, we screened sera from *B*. *pseudomallei*-positive and negative rhesus macaques (Fig 1) using the multiple antigen NHP BurkPx assay for IgM and IgG antibody responses.

**Fig 2:**
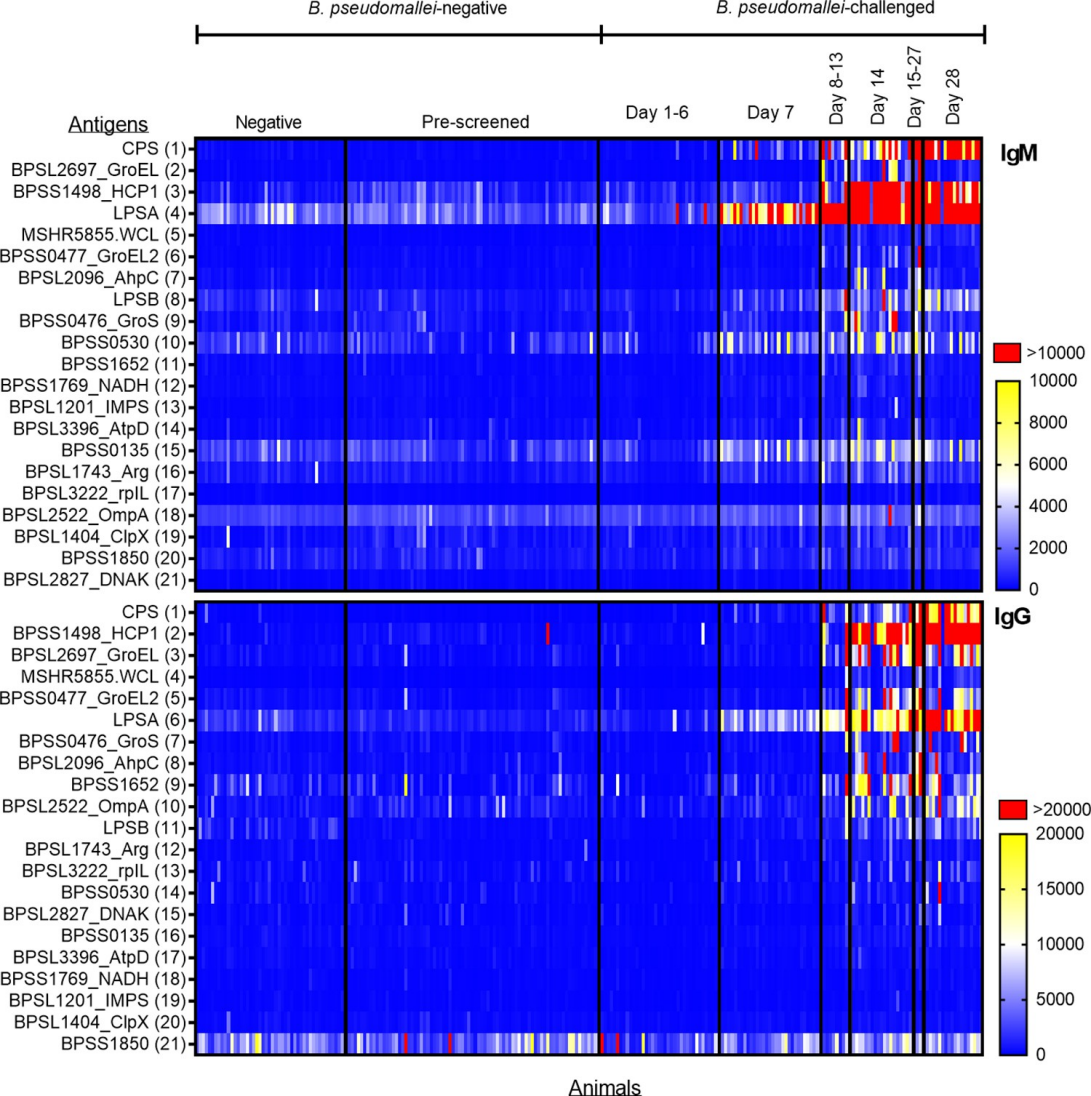
Antigen reactivity for *B*. *pseudomallei*-challenged and *B*. *pseudomallei*-negative NHP sera. Heat maps are shown displaying antigen reactivity for IgM (top panel) and IgG (bottom panel). *Burkholderia* antigens are shown in rows and columns represent individual serum samples. Samples included negative sera (n = 126 sample, n = 126 animals), *B*. *pseudomallei* aerosol challenged sera (n = 115 samples, n = 68 animals) and serum samples were further stratified by prescreening or time after *B*. *pseudomallei* challenge. Antigens were arranged in order by signal to noise ratio and were ordered differently for each isotype. Sera were run in duplicate and the average median fluorescence intensity values (MFI) are shown. IgM ranges were: 0 (blue), 5,000 (white), 10,000 (yellow), and >10,000 (red) as indicated. IgG ranges were: 0 (blue), 10,000 (white), 20,000 (yellow), and >20,000 (red) as indicated.

Early IgM and IgG antibody responses were observed with a 1-fold increase of reactive antibody to LPSA and CPS in samples obtained days 1–6 after challenge compared to the pre-challenge samples (Fig 2). IgM and IgG reactivity to carbohydrates LPSA and CPS were notably increased by Day 7 as well as recognition of LPSB. Reactivity to all three carbohydrates was higher for the IgM response in the first week post infection (IgM fold changes from pre-challenge samples: LPSA 6.0, LPSB 2.1, CPS 13.1; IgG fold changes from pre-challenge samples: LPSA 5.2, LPSB 1.8, CPS 9.2). Serum samples collected during the second week of infection (i.e., days 8–13) exhibited IgG recognition to many *B*. *pseudomallei* proteins with a smaller number recognized by IgM. This trajectory of immune response fits well with the known T-cell independent and dependent activation of B cells. IgM reactivity to HCP1 reached its peak reactivity between days 15–27 with a 25.2-fold MFI change, but then decreased at day 28 to an 11.7-fold MFI change. However, the peak IgG response of HCP1 was observed at day 28 with a 40.5-fold MFI change. Reactivity to proteins GroEL and GroEL2 reached highest levels at day 15–27 but decreased almost entirely for IgM and roughly half for IgG by day 28. Based on a ROC curve analysis, the area under the curve (AUC) was determined. Using simple univariate logistic regression models, the top five antigens for IgM and IgG were CPS (IgM AUC = 0.76, IgG AUC = 0.74, HCP1(IgM AUC = 0.69, IgG AUC = 0.69), WCL (IgM AUC = 0.75, IgG AUC = 0.64), LPSA (IgM AUC = 0.76, IgM AUC = 0.75), LPSB (IgM AUC = 0.67), and OmpA (IgG AUC = 0.62). When compared to a single antigen detection approach, the top five antigens identified by the signal to noise ratio correlate completely for each antibody (Table 2).

**Table 2:** AUC values illustrating the fit of a single antigen model for IgM and IgG BurkPx antigens.

Isotype	Antigen	Week 1	Week 2	Week 3 / 4	All
IgM	CPS	0.6628	0.9713	0.9575	0.7629
IgM	LPSA	0.6118	0.9744	0.9548	0.7566
IgM	WCL	0.6117	0.9636	0.9599	0.7549
IgM	HCP1	0.5215	0.9345	0.981	0.6983
IgM	LPSB	0.5034	0.9342	0.9409	0.6766
IgM	BPSS0135	0.5359	0.9027	0.7911	0.6664
IgM	NADH	0.525	0.8992	0.8097	0.6624
IgM	OmpA	0.5447	0.8641	0.781	0.6608
IgM	BPSS0530	0.5599	0.8351	0.7472	0.6571
IgM	IPMS	0.5232	0.8929	0.7746	0.6537
IgM	Arg	0.512	0.9218	0.8286	0.6491
IgM	GroEL	0.5483	0.9442	0.9167	0.6482
IgM	BPSS1652	0.5516	0.9315	0.8853	0.6378
IgM	GroEL2	0.5424	0.9136	0.8504	0.633
IgM	rpIL	0.5584	0.8623	0.879	0.6165
IgM	AtpD	0.5039	0.851	0.7058	0.6159
IgM	ClpX	0.5122	0.7557	0.6911	0.6005
IgM	GroS	0.5676	0.8392	0.8075	0.5932
IgM	AhpC	0.5843	0.8471	0.8341	0.5898
IgM	DNAK	0.5699	0.7735	0.6706	0.5526
IgM	BPSS1850	0.5743	0.7701	0.7556	0.5369
IgG	LPSA	0.5914	0.9771	0.9698	0.7478
IgG	CPS	0.5909	0.9647	0.9556	0.7421
IgG	HCP1	0.5138	0.9442	0.9806	0.696
IgG	WCL	0.5557	0.9275	0.9452	0.6449
IgG	OmpA	0.5648	0.8304	0.9548	0.6184
IgG	LPSB	0.5819	0.876	0.9115	0.6114
IgG	GroEL	0.6152	0.8824	0.9496	0.599
IgG	Arg	0.6122	0.8657	0.8341	0.5776
IgG	GroS	0.6287	0.8354	0.904	0.5729
IgG	BPSS0135	0.5993	0.8436	0.7919	0.5727
IgG	ClpX	0.5431	0.7424	0.7024	0.5666
IgG	BPSS1652	0.6249	0.8289	0.853	0.5648
IgG	NADH	0.5893	0.7734	0.7982	0.5633
IgG	AhpC	0.6368	0.784	0.8861	0.5529
IgG	GroEL2	0.6559	0.822	0.8556	0.5452
IgG	IPMS	0.5979	0.745	0.6976	0.534
IgG	BPSS0530	0.619	0.7243	0.729	0.5221
IgG	rpIL	0.6286	0.664	0.8143	0.5171
IgG	DNAK	0.6303	0.6571	0.6486	0.5143
IgG	AtpD	0.6468	0.7632	0.7018	0.5101
IgG	BPSS1850	0.6066	0.6042	0.6706	0.5089

In contrast to the *B*. *pseudomallei*-challenged animal serum samples which were pre-screened for *B*. *pseudomallei* antibody reactivity, *B*. *pseudomallei*-negative serum samples from TNPRC appeared to elicit low responses to *B*. *pseudomallei* proteins with intermediate responses to carbohydrates. Due to the differences of the two negative groups, a Mann-Whitney test was performed for all antigens, resulting in statistically significant differences for all carbohydrates, the whole cell lysate and 6/21 proteins for IgG and 8/21 proteins for IgM (p <0.005) (S3 Table). These data suggest that the prescreening adjusted the normal reactivity found in a random population. Furthermore, one pre-challenge NHP sample resulted in a high IgG response to antigen HCP1 (MFI >20,000). This animal was challenged with strain HBPUB10303a at a target dose of 10,000 CFU and died within the first week. The terminal draw before euthanasia had an elevated IgG HCP1 response as well, >10,000 MFI. The preexisting IgG HCP1 reactivity suggests that rare cross-reactivity from other prior infections could exist in rhesus macaques.

Finally, we compared differences between *B*. *pseudomallei* challenge strain or challenge dose. No significant differences in antibody responses for the top five antigens were detected between *B*. *pseudomallei* challenge strain or dose administered. However, the analysis was limited by small sample size caused by early experimental endpoints, as a part of the experimental design of the macaque challenge studies. This preliminary data suggests that within the strains and dosages tested, the immune response will be similar.

### Time-dependent antibody responses

**Fig 3:**
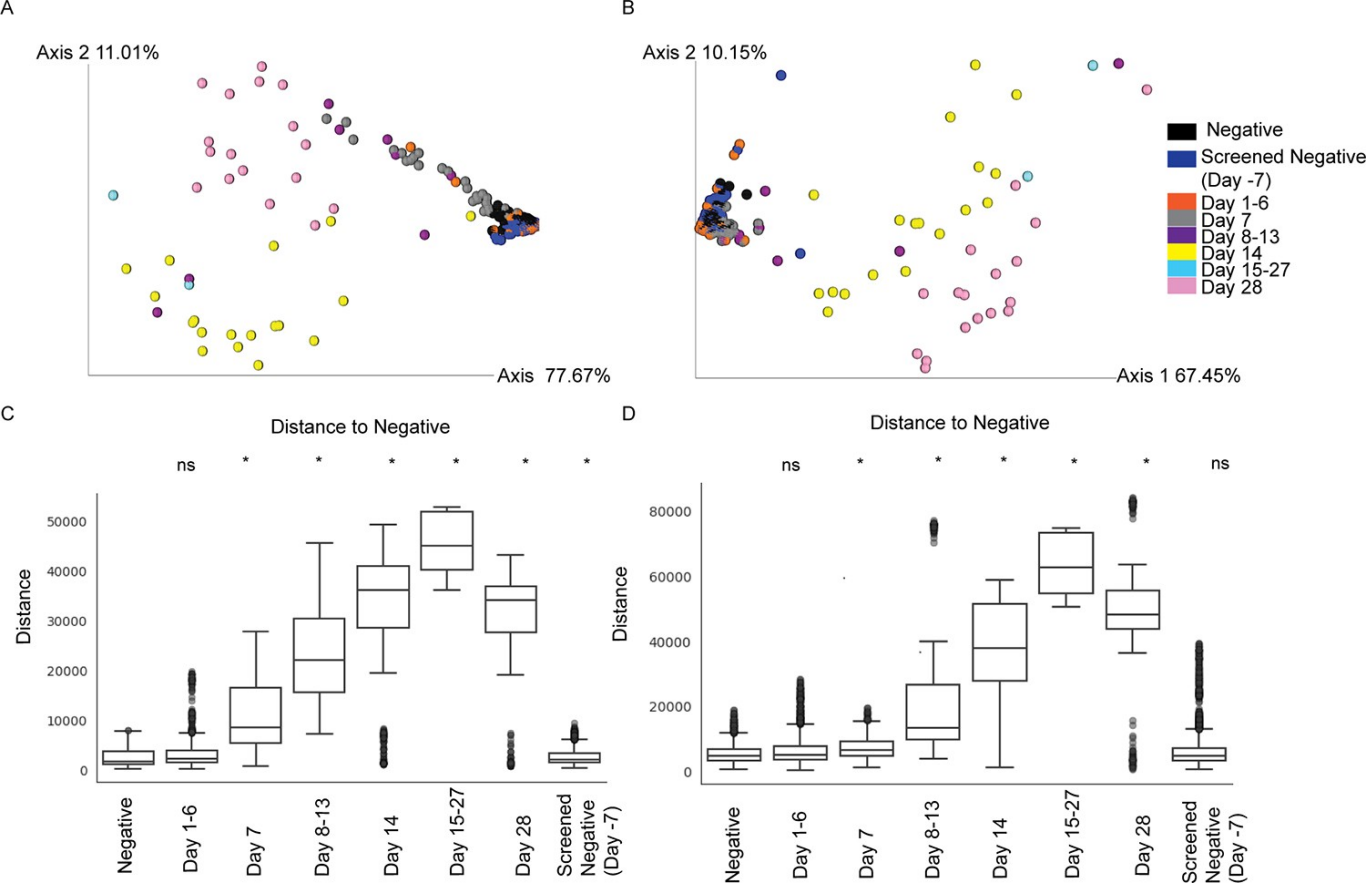
Principal Component Analysis (PCA) and group significance plots of antigen reactive IgM and IgG. Principle Component Analyses (PCA) were performed using Euclidean distance matrices, which were based upon average median fluorescence intensity of all antigens (MFI) independently for IgM (A) and IgG (B) antibodies. The first two principal components were used to plot individual sera in both cases. Permutational multivariate analysis of variance (PERMANOVA) illustrates pairwise distances within and between groups for IgM (C) and IgG (D). “ns” = not significant, * = q <0.005.

A Principal Component Analysis (PCA) was used to determine if the antibody responses to multiple antigens can differentiate serum from *B*. *pseudomallei* exposed and non-exposed animals (Fig 3). For both IgM and IgG, most of the variation is explained by the first component (x-axis) at 77.67% and 67.45% respectively. The second component (y-axis) is responsible for ~10% of the variation in each antibody PCA. In both IgM and IgG analyses, there is a tight clustering of negative (pre-challenge serum samples and negative serum samples) and early *B*. *pseudomallei*-challenged animal samples (day 1–6), suggesting that the Euclidean distance between these samples is rather small. PERMANOVA analysis determined these two groups were not significantly different (q < 0.005) for either antibody isotype (S4 Table). As infection progressed through time, the samples spread out on a continuum along the primary component such that each *B*. *pseudomallei*-challenged animal sample collection time point was significantly different from the pre-challenge animal samples. It is worthwhile to point out the broad spread of early samples in the IgM map, perhaps due to carbohydrate recognition, was observed in the MFI heat maps (Fig 2). This contrasts with the delayed spread of samples until day 14 in the IgG map, indicative of isotype switching. Additionally, the PERMANOVA analysis between pre-challenge samples and negative samples resulted in a significant difference for IgM, but not IgG, potentially lending to the specificity differences of isotypes (S4 Table). This result suggests that IgM antibody has a higher sensitivity to background samples than IgG antibody.

### A multiple antigen model can differentiate infection earlier than single antigens

Based on the results of the PCA, we explored several statistical analysis methods to identify *B*. *pseudomallei*-challenged versus *B*. *pseudomallei*-negative NHP. Ridge Regression and Least Absolute Shrinkage and Selection Operator (LASSO) models were applied for IgM, IgG, or a combination of IgM and IgG. All models were initially trained using cross-validation on a randomly chosen naïve dataset and then validated with new data to evaluate performance (S1 Fig). Each model was evaluated using independent data and subsequent ROC analysis was performed to determine AUC values. In the validation assessment, the IgGM Lasso model had the highest Week 1 AUC Value (0.85), followed by the IgG Lasso model (week 1 AUC 0.83). When comparing the AUC values of all time points combined, the IgGM Lasso and IgGM Ridge models performed equally with an AUC of 0.91, higher than any of the single antigen model AUC values for IgM and IgG (Table 2).

**Fig 4:**
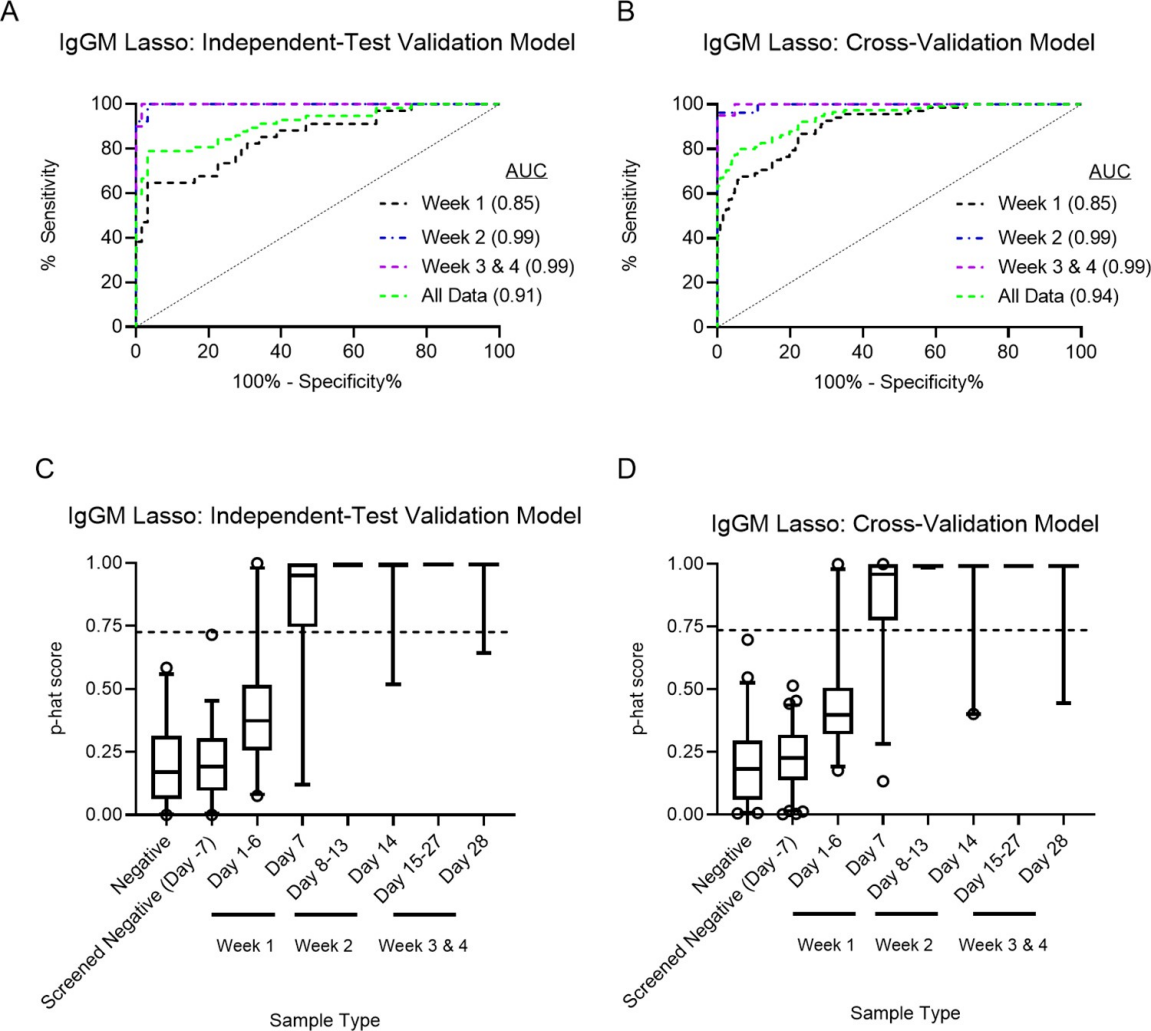
Receiver Operator Characteristic (ROC) plots and box and whisker plots of the IgGM Lasso multiple antigen models. $\hat{p}$-scores (p-hat) were generated from median fluorescent intensity values of all antigens for IgM and IgG. (A and C) Independent test validation and (B and D) cross-validation performance LASSO models are shown.

A second model was trained using cross-validation on all the data (S2 Fig). We observed similar cross-validation performance metrics as the independent data validation of the first model, with the IgGM LASSO performing best at week 1 (AUC value 0.85) and the IgGM LASSO and IgGM Ridge Regression performing equally well when all time points are combined (AUC value 0.94). To further analyze the models, box and whisker plots were generated with p-hat scores plotted for each sample, separated by time points (S3 Fig). An arbitrary cutoff value was established for each model by applying Youden’s J-score to determine a sensitivity value while maintaining 100% specificity. The sensitivity levels of the two highest performing applied models, IgGM LASSO and IgGM Ridge Regression were 41.2% and 35.3% at week 1 and 63.48% and 60% when all time points were combined (S5 Table). Thus, due to the higher sensitivity at week 1 and when all time points were combined, we deemed the IgGM LASSO model to be the best performer for early detection of *B*. *pseudomallei* exposed NHP (Fig 4).

## Discussion

NHP are one of the few animal species that are ideal models for melioidosis research due to their translational relevance to clinical melioidosis in humans [20]. However, since NHP are also susceptible to natural infection with *B*. *pseudomallei*, concerns of a pre-existing immunity emerge. A diagnostic test to detect *B*. *pseudomallei* exposures in NHP is a necessary tool to monitor sporadic outbreaks and ensure NHP enrolled in research are free of current or past infection. In the present study, we used the findings of the antigen evaluation completed by [25] to formally evaluate a diagnostic test, the BurkPx assay, for detection of *B*. *pseudomallei* exposure for NHP. Our goals were two-fold. The first was to standardize this melioidosis diagnostic test for NHP and the second was that by using a previously developed assay based on a set of highly diagnostic human-based antigens, we could identify additional uses of the diagnostic tool with respect to vaccine research.

All 21 immunoreactive antigens identified in [25] with the highest diagnostic potential for detecting melioidosis in humans were employed for detection of *B*. *pseudomallei* exposure in NHP. Our use of xMAP technology to develop a multiplex MAGPIX assay was chosen due the ability of this technology to measure multiple analytes in a single sample, shorter turnaround time, and strong track record in sensitivity and specificity for detection of a variety of pathogens [34]. Evaluation of our NHP BurkPx assay with sera taken from two sources–sera from defined timepoints after NHP aerosol challenge with *B*. *pseudomallei* for determining LD_50_ values and sera from healthy NHP–produced an overall sensitivity of 63.48% and specificity of 100%. When the time-points were split, sera collected one week post challenge contributed to the reduced overall sensitivity value. After week two, three, and four post challenge, the sensitivity was 95% or greater. While symptoms of *B*. *pseudomallei* infection generally require about four days to develop in humans, we have observed low sensitivity within the first week of infection which may be explained by detection limits of antibodies (as in, the antibody levels are not high enough for detection). This limitation leads us to two testing recommendations: One, NHP should be screened 2 weeks after removal from outdoor housing when intended for enrollment in preventative medicine and population screening; Two, NHP with symptoms of disease or other contributing factors should be serial tested over time.

The sample set used provided the opportunity to evaluate antigen responses across time, antibody type, and sample type. Not surprisingly, the IgM and IgG immune responses in NHP followed a similar seroconversion time that is well-known in humans. This was evident in the PCA and subsequent PERMANOVA analysis. Carbohydrates, namely CPS and LPSA, elicited high IgM responses and mediocre IgG responses early in the infection. Carbohydrates are commonly the first antigen type detected by the humoral immune response since they are surface molecules and can be directly recognized by B-cells [35]. As infection progressed, we began to observe high signals of prominent antigens detected by the human immune system like GroEL, GroEL2, CPS, LPSA, and HCP1. These five antigens have been employed in a variety of human diagnostic tests including a protein microarray [36], a rapid lateral flow assay [37], and various ELISA assays [38,39], just to name a few, demonstrating consistency of this multiplexed assay. The dominant LPSA response could be explained by the origin of strains used for the challenge. In humans, there is a geographic correlation between LPSA and LPSB responses [40].

Unfortunately, due to sample size, no statistical analysis could be conducted to determine if there were any antibody response differences caused by infective dose or different strains. However, we believe that the variety in strains and doses administered in the LD_50_ study in conjunction with the background history of both NHP sample sets has increased our assay robustness in multiple antigen model building overall. The *B*. *pseudomallei*-negative NHP serum samples from TNPRC were collected after the accidental *B*. *pseudomallei* release and routinely tested for *B*. *pseudomallei* antibody reactivity, supporting the low background results we observed, even though roughly half of these NHP were born before the outbreak event (Fig 2). Moreover, *B*. *pseudomallei*-challenged NHP were of Chinese origin, but pre-screened for *B*. *pseudomallei* antibody reactivity. We did observe one NHP with an elevated IgG antibody response to HCP1 in its pre-screened/pre-challenge serum that decreased slightly in signal in the post-challenge/terminal draw. We repeatedly tested the pre-challenge/pre-screened sample to confirm the unusual response and can only reason that this event may be due to rare HCP1 cross reactivity with other related bacteria or prior exposure to *B*. *pseudomallei* that the NHP may have experienced in the past. It is important to note the pre-screening test employed for existing *B*. *pseudomallei* antibodies, an IgG *B*. *pseudomallei* WCL ELISA, would not have captured pre-existing HCP1 reactivity due to the media the *B*. *pseudomallei* was cultured in [41]. Moreover, we speculate that the slight reduction in IgG HCP1 reactivity at the animals’ terminal draw (Day 1–6) may be due to time post infection or a competition of a separate untested (i.e., IgA) antibodies. Moreover, we are aware that this sample set only included animals that were aerosol challenged, and thus, the diagnostic test evaluated here and resulting cut-off values may only be applicable to NHP suspected of aerosol exposure.

Defining cutoff values for detection of any pathogen requires an assessment of the tests intended use. The *B*. *pseudomallei* diagnostic field has been perpetually challenged by background serological responses due to endemicity or other Gram-negative bacterial exposure. We addressed this issue by including *B*. *pseudomallei* naïve NHP serum samples into the sample set and training of the multiple antigen model. This model type produces a p-hat score (i.e., proscore), a simple binary score that can be applied with a cutoff value to determine likelihood of exposure. Currently, the cut-off value of our model was based upon the highest sensitivity value when specificity was maintained at 100%. This threshold value resembles one that might apply when identifying the source of a melioidosis outbreak. In this case, an outbreak may consist of an environmental source, a bioweapons event, or an accidental release where false positives would greatly hinder the user’s ability to identify to the source. As such, this circumstance would define cutoffs that result in high specificity and lower sensitivity. In contrast, when diagnosing a patient with melioidosis, the false negative rate would be of highest concern, so cutoff values may be defined to maximize sensitivity with reduced specificity. This criterion may result in false-positives and lend to non-melioidosis diseased patients being treated with unneeded antibiotics, but secondary confirmatory tests may reduce this risk. When using this tool for purposes of population surveillance to understand natural exposure rates, the endemic or non-endemic regional origins of the samples must be taken under consideration. In an endemic region, historical data might be used to define cutoffs, then adjusted as serial sample data reveals increasing or decreasing trends. Further information may be gathered by conducting comparative tests, such as looking at endemic versus non-endemic rates and adjusting cutoff values to address the research questions.

The relationship proposed between melioidosis vaccine trials and our NHP BurkPx assay lies in the potential for detecting correlates of protection (COPs). There is a growing interest to use NHP in melioidosis clinical trials due to the guidelines by the FDA and SMVTD groups. Nonetheless, the interest of using the NHP BurkPx assay to detect immunological responses of vaccinated NHP has already been beneficial. We recognize that there are two levels of COPs in *B*. *pseudomallei* infections, antibody and cell-mediated responses, and our assay only addresses antibody-based COPs [18]. Fortunately, identifying immunological COPs with our NHP BurkPx assay can be directly relatable to humans because this assay was built upon human diagnostic antigens.

In summary, we evaluated a serological multiplex assay for the detection of *B*. *pseudomallei* exposure in NHP and believe it is the first extensively evaluated diagnostic tool for use with NHP. Our technical approach offers quantitative and qualitative data collection for twenty-one antigens in a single assay. Some of the twenty-one antigens were not as effective for detection in NHP as they were for detecting melioidosis in humans. This approach reduced time and effort for building a new assay, allows for direct translation between hosts, and will increase its applicability in vaccine studies. The NHP BurkPx assay is expected to be useful for surveillance in NHP colonies, in investigations of suspected accidental releases or exposures, and for identifying vaccine correlates of protection. To broaden the spectrum of this assay, future evaluations can be conducted on larger NHP serum sample sets from multiple species and with various modes of infection, exposure, doses, and strains. We acknowledge the limited sample set and restriction to inhalational samples, but trust that the assay’s strength lies in the translational approach we took by using human antigens.

## Supporting information

S1 FigReceiver Operator Characteristic (ROC) plots of Independent-Test validation multiple antigen models.$\hat{p}$-scores were generated from median fluorescent intensity values of all antigens for IgM only, IgG only, or a combination of IgGM. The evaluated model was trained on half the data and tested with a non-modeled dataset. Area under the curve (AUC) values are identified for each line, indicating the probability of the assay correctly defining a positive sample. The dashed line along the diagonal signifies the result of a random assay.(TIF)

S2 FigReceiver Operator Characteristic (ROC) plots of Cross-Validation multiple antigen models.$\hat{p}$-scores were generated from median fluorescent intensity values of all antigens for IgM only, IgG only, or a combination of IgGM. Shown here is the cross-validation performance of the model when trained with all collected data. Area under the curve (AUC) values are identified for each line, indicating the probability of the assay correctly calling a positive sample. The dashed line along the diagonal signifies the result of a random assay.(TIF)

S3 Fig$\hat{p}$-score (p-hat) box and whisker plots for Cross-Validation multiple antigen models.Six multiple antigen models were evaluated for best performance, indicated by high sensitivity and a specificity set at 100%. Graph (a) is the best performing model. Models in (B–F) are the sub-optimal models with cutoff thresholds defined by Youden’s J-score with a bias to maintain specificity at 100%.(TIF)

S1 Table *Burkholderia pseudomallei* aerosol challenge results. (XLSX)

S2 Table IgGM Lasso Cross-Validation Model coefficients. (XLSX)

S3 Table Mann Whitney T-test results of negative serum samples compared to *B*. *pseudomallei*-screened negative serum samples. (XLSX)

S4 Table Pairwise PERMANOVA statistical results. (XLSX)

S5 Table Summary statistics for ROC analysis of multiple antigen models. (XLSX)

S6 Table Final Median Fluorescence Intensity (MFI) values from NHP serum samples. (XLSX)
