# Supplementary figures and images for: NHP BurkPx: A multiplex serodiagnostic bead assay to monitor Burkholderia pseudomallei exposures in non-human primates
Source: PLoS Negl Trop Dis. 2023 Feb 8;17(2):e0011067. doi: 10.1371/journal.pntd.0011067 (PMC9907805; doi:10.1371/journal.pntd.0011067)

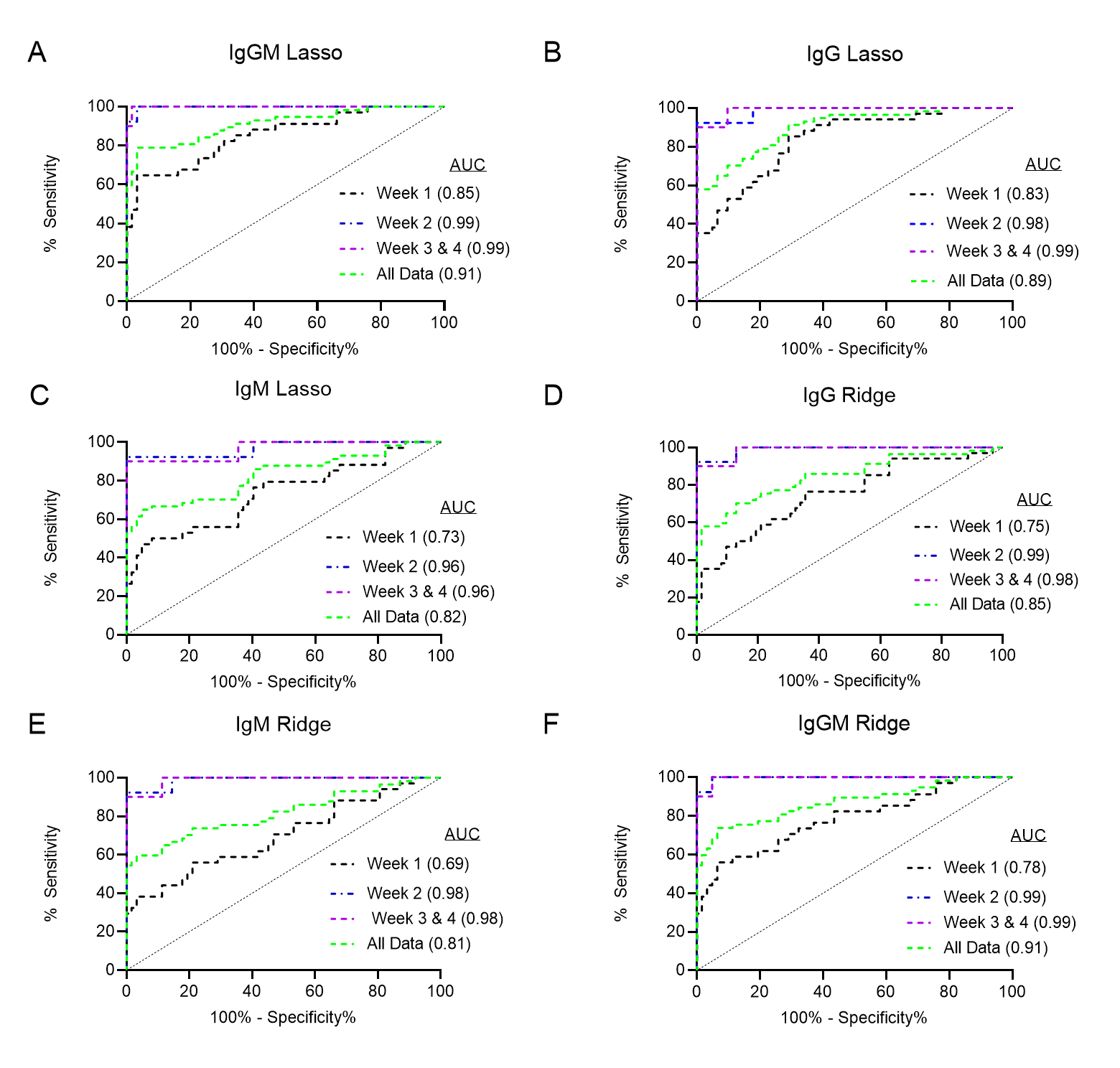

Supplement: S1 Fig — p^-scores were generated from median fluorescent intensity values of all antigens for IgM only, IgG only, or a combination of IgGM. The evaluated model was trained on half the data and tested with a non-modeled dataset. Area under the curve (AUC) values are identified for each line, indicating the probability of the assay correctly defining a positive sample. The dashed line along the diagonal signifies the result of a random assay. (TIF) [file pntd.0011067.s001.tif]

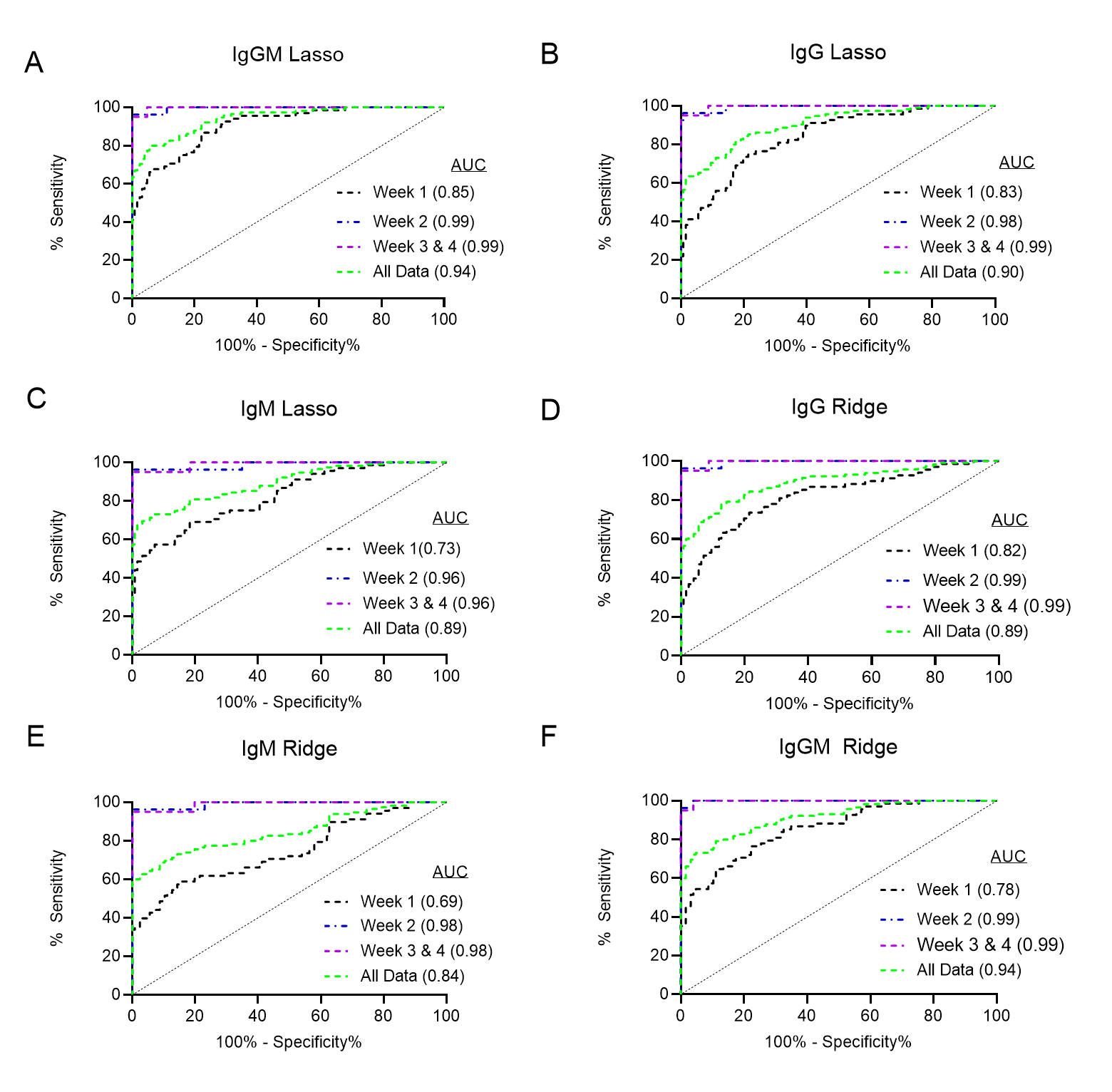

Supplement: S2 Fig — p^-scores were generated from median fluorescent intensity values of all antigens for IgM only, IgG only, or a combination of IgGM. Shown here is the cross-validation performance of the model when trained with all collected data. Area under the curve (AUC) values are identified for each line, indicating the probability of the assay correctly calling a positive sample. The dashed line along the diagonal signifies the result of a random assay. (TIF) [file pntd.0011067.s002.tif]

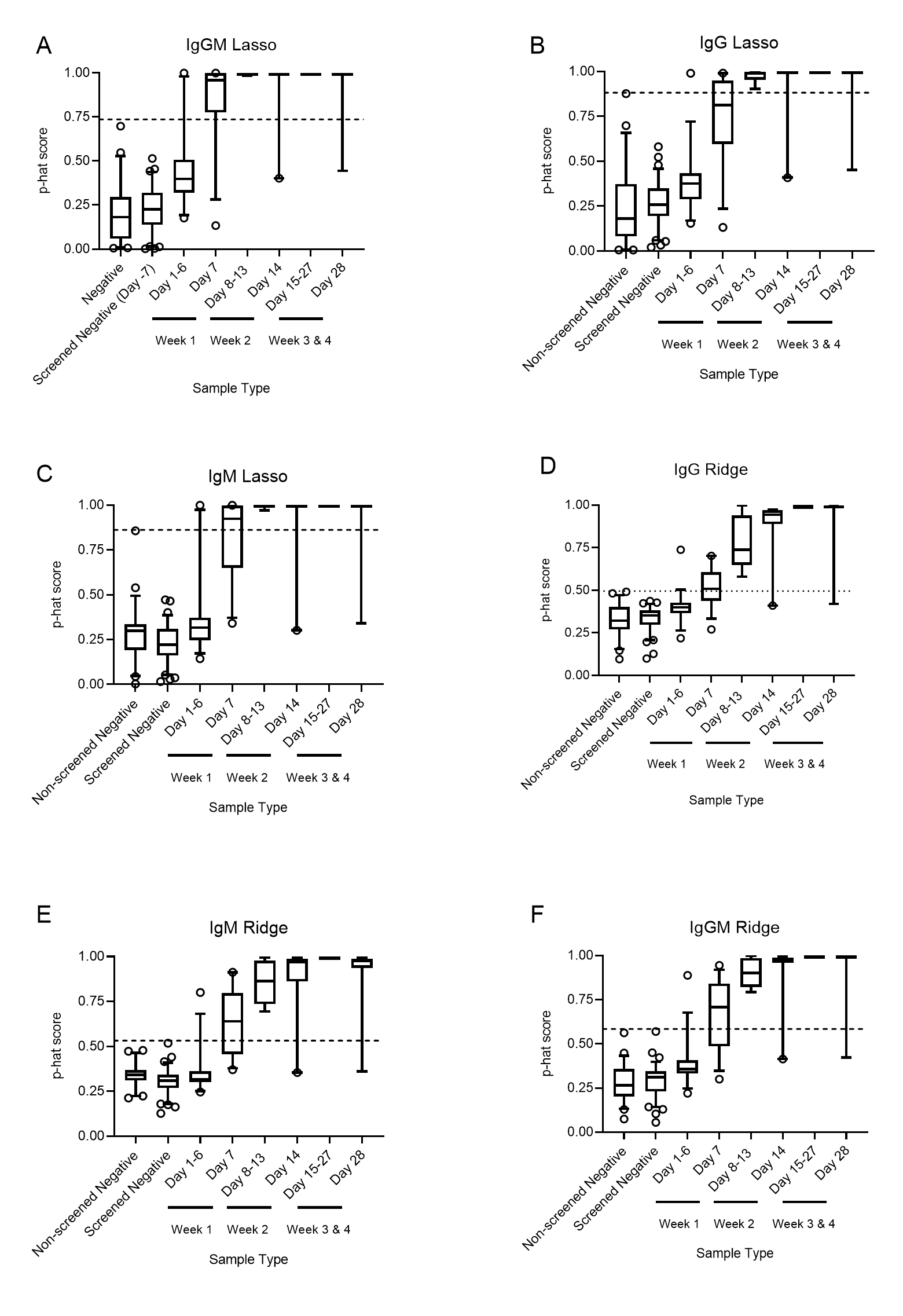

Supplement: S3 Fig — Six multiple antigen models were evaluated for best performance, indicated by high sensitivity and a specificity set at 100%. Graph (a) is the best performing model. Models in (B–F) are the sub-optimal models with cutoff thresholds defined by Youden’s J-score with a bias to maintain specificity at 100%. (TIF) [file pntd.0011067.s003.tif]
